# Clinical efficacy of percutaneous endoscopic posterior lumbar interbody fusion and modified posterior lumbar interbody fusion in the treatment of lumbar degenerative disease

**DOI:** 10.1186/s13018-024-04544-y

**Published:** 2024-01-16

**Authors:** Zhengping Liu, Siyu Wang, Tao Li, Si Chen, Ying Li, Wei Xie, Jin Tang

**Affiliations:** 1 School of Sports Medicine, Wuhan Sports University, Wuhan, Hubei China; 2 Department of Minimally Invasive Spinal Surgery, The Affiliated Hospital of Wuhan Sports University, No 279 Luoyu Road, Hongshan District, Wuhan, 430079 Hubei China

**Keywords:** Percutaneous endoscopic posterior lumbar interbody fusion, Modified posterior lumbar interbody fusion, Lumbar degenerative disease, Clinical outcome

## Abstract

**Background:**

To compare the early clinical efficacy of percutaneous endoscopic posterior lumbar interbody fusion (PE-PLIF) and modified posterior lumbar interbody fusion (MPLIF) in the treatment of lumbar degenerative disease (LDD).

**Methods:**

A total of 37 patients who underwent PE-PLIF and 58 patients who underwent MPLIF from March 2019 to January 2022 were retrospectively reviewed. The operation time, intraoperative blood loss, post-operative hospitalization time, and post-operative bedrest time were recorded. The visual analogue scale (VAS) scores of leg pain and low back pain, Japanese Orthopaedic Association (JOA) scores, and the Oswestry Disability Index (ODI) scores were evaluated and compared before the operation, 3 days after the operation, 1 week after the operation, 1 month after the operation, 6 months after the operation and at the last follow-up. The modified MacNab’s criteria were applied at the last follow-up. The fusion rate and surgical-related complications during follow-up were recorded.

**Results:**

The average operation time in the PE-PLIF group was highly significant longer than that in the MPLIF group (*P* < 0.01). The intraoperative blood loss, post-operative hospitalization time, and post-operative bedrest time were significantly less in the PE-PLIF group than those in the MPLIF group (*P* < 0.01). There were highly significant differences in VAS scores of leg pain, VAS scores of low back pain, JOA scores, ODI scores at the last follow-up compared with those before the operation in the two groups (*P* < 0.01). Three days after the operation and 1 week after the operation, the VAS scores for low back pain and ODI were highly significant less in the PE-PLIF group than that in the MPLIF group (*P* < 0.01). Three days after the operation, the JOA scores were highly significant higher in the PE-PLIF group than that in the MPLIF group (*P* < 0.01). All patients showed intervertebral fusion at 6 months after the operation. Two patients (5.4%) in the PE-PLIF group experienced complications.

**Conclusion:**

Both PE-PLIF and MPLIF surgery were clinically effective and safe for patients with single-segment LDD. PE-PLIF surgery is a promising technique that can be used as an alternative treatment for single-segment LDD.

## Background

Lumbar degenerative disease (LDD) is a general term for a series of diseases with clinical symptoms such as low back pain and leg pain, and it is caused by degenerative changes in the lumbar intervertebral disc, articular process, and ligaments.

Lumbar discectomy and lumbar interbody fusion (LIF) are the main treatment methods for LDD when conservative treatment is ineffective [1]. LIF is an important method to achieve complete decompression and rebuild spinal function. The main indications for LIF include lumbar spinal stenosis (LSS), lumbar spondylolisthesis (LS), lumbar disc herniation, and scoliosis. LIF is widely used in the clinic because it can alleviate pain, relieve nerve root compression, redress lordosis, and correct spinal deformity. Posterior lumbar interbody fusion (PLIF) is a well-established technique with definite efficacy for treating LDD. Traditional PLIF, however, necessitates extensive removal of the vertebral lamina, spinous process, supraspinous ligament, interspinous ligament, ligamentum flavum, and facet joints, which damages the posterior spinal ligament complex, compromises the stability of the spine, and increases the risk of adjacent segment degeneration (ASD) [2]. The preservation of the posterior spinal ligament complex can maintain the stability and flexibility of the spine, maintain the physiological and mechanical function of the spine, and prevent the occurrence of ASD after PLIF [2, 3].

Modified posterior interbody fusion (MPLIF) reduces damage to the normal spinal structure and retains the spinous process and posterior spinal ligament complex, with good clinical efficacy [3]. Therefore, we performed MPLIF to treat patients with LDD. With the development of minimally invasive techniques, an increasing number of surgeons have performed percutaneous endoscopic lumbar interbody fusion (PE-LIF) to treat patients with LDD. PE-LIF is advantageous in that it causes in little muscle injury and facilitates fast recovery and making it a promising treatment for LDD [4–6]. Percutaneous endoscopic posterior lumbar interbody fusion (PE-PLIF) is a common clinical treatment method for LDD. Currently, there are few studies comparing the clinical efficacy of PE-PLIF with that of MPLIF in the treatment of LDD. In this study, we analysed the perioperative parameters and clinical and radiological outcomes of PE-PLIF and MPLIF to evaluate the early clinical efficacy of PE-PLIF and MPLIF in the treatment of LDD.

## Materials and methods

### General data

For this retrospective cohort study, we collected the clinical data of patients who underwent PE-PLIF surgery and MPLIF surgery for LDD in our hospital from March 2019 to January 2022. PE-PLIF surgery was performed on the patients in the PE-PLIF group (37 cases), and MPLIF surgery was performed on the patients in the MPLIF group (58 cases). There were no significant differences in sex, age, disease type, surgical segment, or follow-up time between the two groups (*P* > 0.05, Table 1). All patients signed informed consent forms. All procedures were performed by the same group of senior spine surgeons with extensive experience in endoscopic and open surgery. The study was approved by the hospital ethics committee.

**Table 1:** General data of patients in PE-PLIF group and MPLIF group

	PE-PLIF (*n* = 37)	MPLIF(*n* = 58)	*P*
Sex (Male/Female)	22/15	26/32	0.164
Age (years)	56 (51.5, 63)	58.5 (51, 65.25)	0.691
Disease type			
LS	26	30	0.073
LSS	11	28	
Surgical level			
L3/4	8	7	0.461
L4/5	16	28	
L5/S1	13	23	
Follow-up time (months)	15 (13.5, 16)	15 (14,16)	0.576

**Fig. 1:**
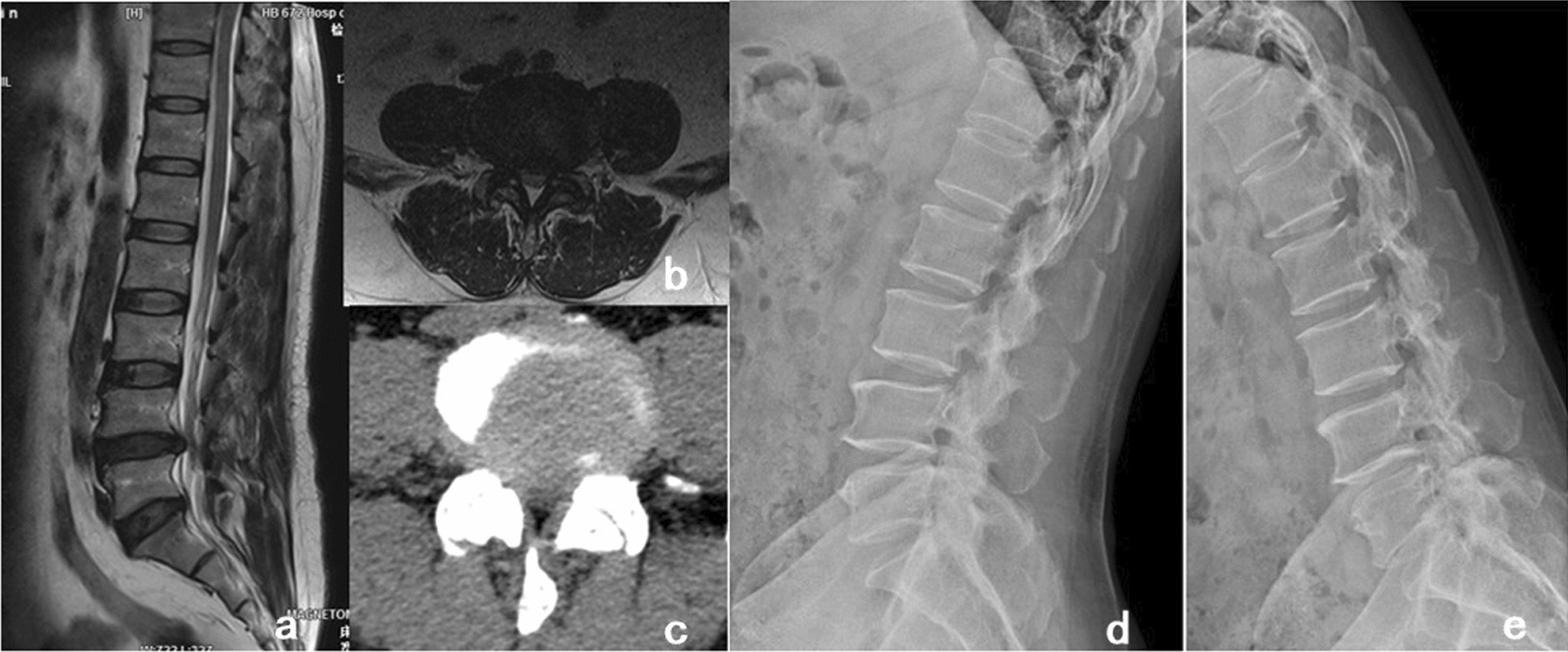
**a**, **b** Pre-operative MRI in sagittal and coronal positions showed a herniated disc at L4/5 with the hyperplastic ligamentum flavum; **c** Pre-operative CT in coronal position showed a herniated disc at L4/5 with the narrow spinal canal; **d**, **e** Pre-operative dynamic X-ray radiographs showed the stability of lumbar spine was good

Inclusion criteria:

1. Patients diagnosed with single-segment LDD, including LS (Meyerding ≤ II), LSS, with or without disc herniation, whose symptoms and signs were consistent on lumbar X-ray, computed tomography (CT) images, and magnetic resonance imaging (MRI) (Fig. 1);
2. Patients with persistent neurological symptoms or typical intermittent claudication symptoms;
3. Patients with symptoms that could not be alleviated or were aggravated at least 3 months after nonsurgical treatment;
4. Patients who underwent PE-PLIF or MPLIF surgery.

Exclusion criteria:

1. Patients with obvious scoliosis or kyphosis;
2. Patients with severe heart, brain, kidney, or other types of disease who could not tolerate the surgery;
3. Patients with a history of lumbar surgery;
4. Patients with severe osteoporosis;
5. Patients with lumbar tumour, tuberculosis, or infection;
6. Patients themselves or whose family did not consent to the study;
7. Patients with psychological disorders or mental disorders.

### Surgical methods

1. PE-PLIF

**Fig. 2:**
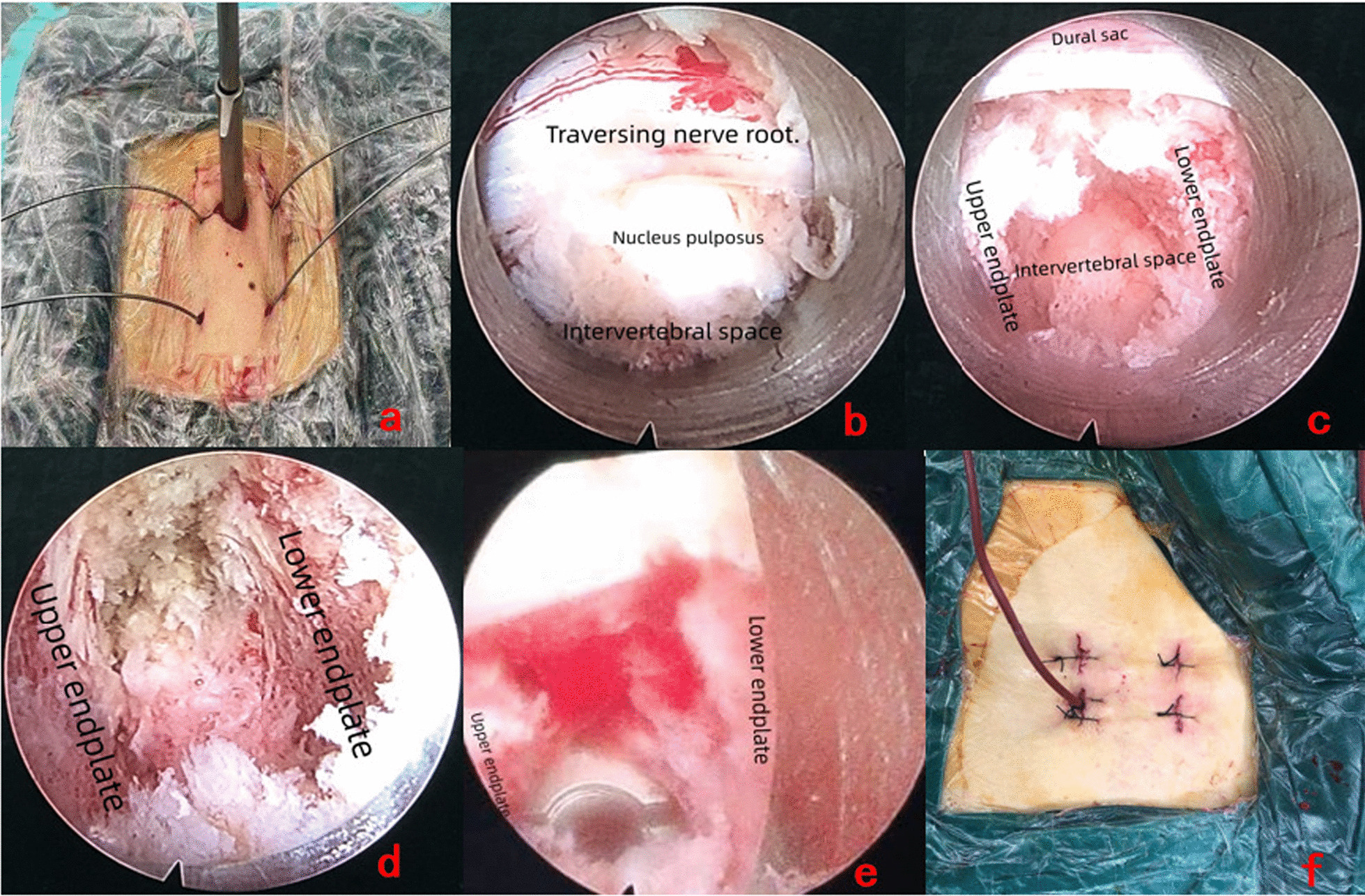
**a** Guide wire and channel were successfully inserted; **b**–**e** The upper endplate, lower endplate, nerve root, dural sac, intervertebral space, and cage were seen under the endoscope; **f** The condition of post-operative wound and drainage tube

All operations were completed under general anaesthesia. Patients were in the prone position. The table was adjusted to moderately flex the lumbar and expand the intervertebral space appropriately. The surgical level was confirmed under C-arm fluoroscopy prior to the operation. The surgical area was routinely disinfection and draped. The skin incision was located next to the targeted intervertebral space, approximately 2 cm away from the spinous process. A longitudinal incision of approximately 15 mm was made. The incision was gradually expanded with soft tissue dilatation tubes to allow insertion of the working channel. A large channel spinal endoscopy system (Unintech system, Joimax, Germany) was used in the surgery. In the surgical section, anatomical structures such as the upper and lower vertebral laminas, articular process, and ligamentum flavum were revealed sequentially under the endoscope with straight forceps, curved forceps, and radiofrequency ablation electrode (APS-A-01-N-7030/ Aceso-Suzhou). Bony structures such as the superior vertebral body portion of the vertebral lamina, inferior articular process (IAP), lateral recess, and inferior vertebral body portion of the vertebral lamina in the operative area were sequentially removed by using a visual trephine with an endoscopic bone knife until the origin and end of the ligamentum flavum were revealed. The lateral wall of the superior articular process (SAP) was preserved to protect the exiting nerve roots. The ligamentum flavum was partially excised to expose the nerve roots, dural sacs, and intervertebral disc. The working channel was rotated so that its bevel moved towards the lateral side to prevent nerve roots from entering the working space. Nucleus forceps were used to extract the nucleus pulposus, and reamers and scrapers were used to trim the cartilaginous endplate. After the endplates were completely prepared, autologous bone and allograft bone (Aoli, Shanxi, OSTEORAD Biomaterial Co., Ltd. Taiyuan), recombinant human bone morphogenetic protein-2 (rhBMP-2) (Hangzhou Jiuyuan, rhBMP-2, Hangzhou Jiuyuan Gene Engineering Co., Ltd.), and an expandable cage (Shanghai Reach Medical Instrument Co., Ltd.) were implanted into the intervertebral space. After decompression of the spinal canal confirmation that the cage was in a satisfactory position under fluoroscopy and endoscopy, the endoscope and working channel were withdrawn. Finally, percutaneous pedicle screws (RS8 LONG Long Tail Minimally Invasive System, Shanghai Reach Medical Instrument Co., Ltd.) and connecting rods were implanted, and the skin incision was sutured, but a drainage tube was not placed. However, a drainage tube was placed in case of excessive bleeding. (After decompression, the normal saline perfusion was turned off, and all the blood oozed under the endoscope.) The drainage tube could be removed 1–2 days after the operation depending on the amount of drainage [7]. The incision was bandaged to end the operation. (Fig. 2).

2. MPLIF

All operations were completed under general anaesthesia. Patients were in the prone position. The table was adjusted to moderately fix the lumbar spine. Routine disinfection and towel laying were performed. A longitudinal incision was made along the midline of the spinous process at the surgical section. The spinous process and posterior spinal ligament complex were preserved. The bilateral paraspinal muscles were dissociated from the subperiosteum and exposed to the lateral margin of the articular process. Pedicle screws were inserted into the superior outer edge of the apex of the “∧” shaped crest. After the pedicle screws were placed in a satisfactory position, decompression was performed on the affected side (unilateral or bilateral). The parts of the upper and lower vertebral laminas, approximately 1/3 of the IAP, SAP, and ligamentum flavum were removed. The dural sacs and nerve roots were protected, and the intervertebral space was treated until cartilaginous endplate bleeding was punctate. After the endplates were completely prepared, the bone grafts and a suitable cage were inserted into the intervertebral space. Bilateral connecting rods were installed and fixed with appropriate pressure. The wound was sutured after complete haemostasis and catheter drainage.

### Post-operative treatment

**Fig. 3:**
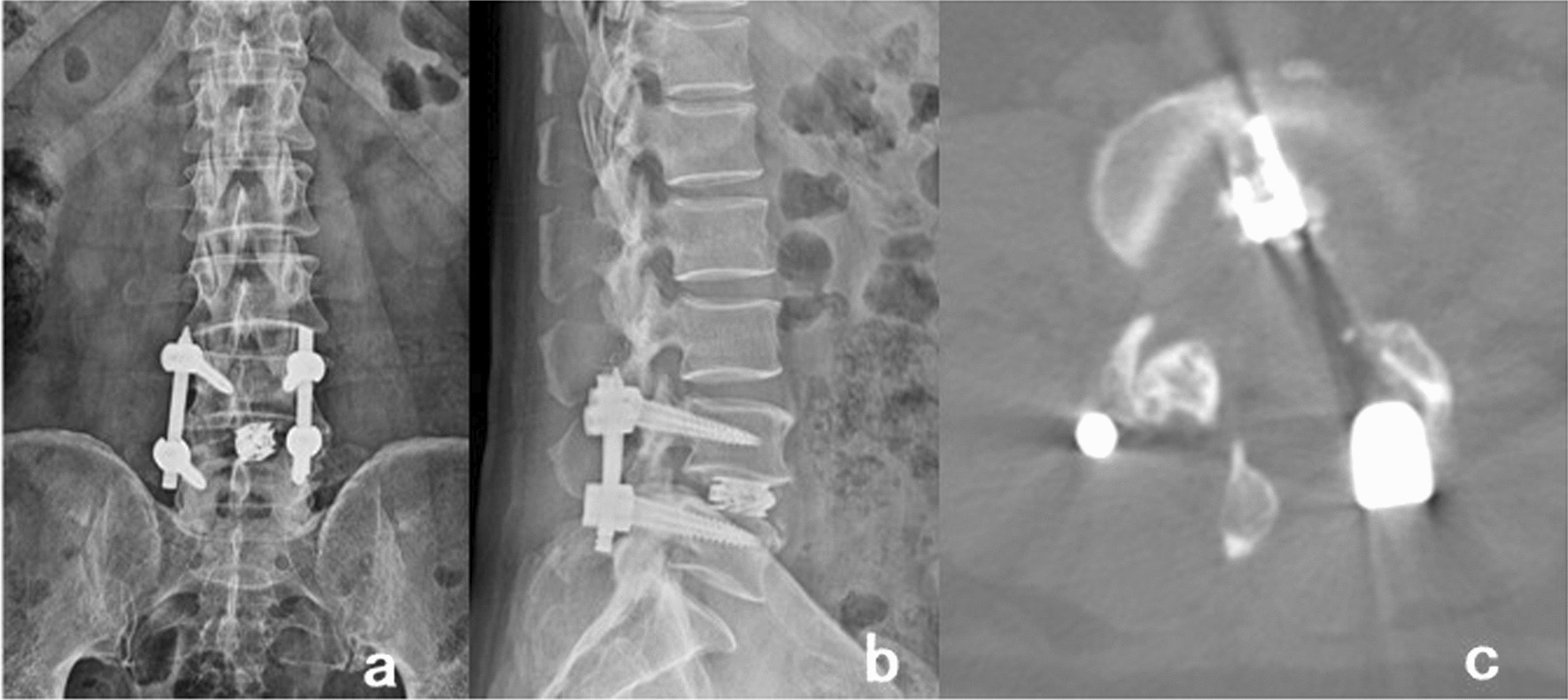
**a**, **b** Post-operative anteroposterior and lateral X-ray radiographs showed the pedicle screws, and the cage were in good position; **c** Post-operative CT in coronal position showed that the cage was in good position, and the cross-sectional area of the spinal canal was significantly enlarged compared with that before operation

After surgery, patients in both groups were placed on bedrest and received the same rehydration regimen, including painkillers. Patients were in bed for 1–2 days in the PE-PILF and 4–5 days in the MPLIF group. According to Caprini scores, appropriate regimens of thrombosis prevention were chosen for patients [8]. All patients were encouraged to turn over in bed and lift their legs while receiving leg pressure therapy to prevent thrombosis. Patients without a drainage tube were by re-examined lumbar X-ray and CT on the first day after surgery, while patients with the placement of a drainage tube were re-examined by lumbar X-ray and CT on the day on which drainage tubes were removed (Fig. 3). If the internal fixation and cage position were satisfactory, the patients got out of bed with the assistance of a lumbar brace.

### Observation index

1. Surgical-related index

The surgical-related outcomes of the two groups were recorded, mainly including the operation time, intraoperative blood loss, post-operative hospitalization time, post-operative bedrest time, and complications.

2. Clinical efficacy index

All patients were evaluated with visual analogue scale (VAS) scores, Japanese Orthopaedic Association (JOA) scores, and Oswestry Disability Index (ODI) scores preoperatively (1 day prior to the operation), as well as 3 days, 1 week, 1 month, and 6 months after the operation and at the last follow-up. The modified MacNab’s criteria were applied at the last follow-up.

VAS scores for low back pain and leg pain were recorded, with scores ranging from 0 to 10 points. A higher score indicated more severe pain [9].

The JOA scores were recorded to assess neurological function, with scores ranging from 0 to 29 points. A higher score indicated more significant improvement of neurological function [9].

The ODI scores were recorded to assess physical dysfunction, with a total score of 50 points. A higher score indicated more serious physical dysfunction and worse quality of life [10].

The modified MacNab’s criteria were applied to assess the treatment outcomes of the patients [9]. The criteria have four grades. Excellent: Symptoms disappeared completely, successfully returning to work and life. Good: Slight symptoms, slightly limited activities, no effect on work or life. Fair: Symptoms were relieved, but activities were limited, affecting normal work and life. Poor: There was no difference in the perioperative period, symptoms may even be worse.

3. Radiological evaluation index

Lumbar anteroposterior, lateral, and dynamic X-ray radiographs were collected before the operation. Lumbar anteroposterior and lateral X-ray radiographs were collected at 1 month, 3 months, and 6 months postoperatively. The final fusion grade was assessed 6 months after surgery using the Bridwell criteria on CT images [11]. Grade I, fused with remodelling and trabeculae present; Grade II, graft intact, not fully remodelled and incorporated, but no lucency present; Grade III, graft intact, potential lucency present at the top and bottom of the graft; Grade IV, fusion absent with collapse/resorption of the graft.

### Statistical analysis

All statistical analyses were performed using SPSS 27.0. General data were described statistically, and data that did not conform to the normal distribution were represented by the median (first quartile, third quartile). The Mann‒Whitney U test was used to compare the differences between the two groups, and the Friedman test was used to compare the groups at different time points and multiple comparisons. Categorical variables were expressed as frequencies or percentages and were compared with the Chi-squared test or Fisher's exact test. A *P* value < 0.05 was considered to indicate statistical significance, and a *P* value < 0.01 was deemed to indicate highly significant.

## Results

### Surgery-related index

In the PE-PLIF group, the average operation time was significantly longer than that in the MPLIF group (*P* < 0.01). The intraoperative blood loss in the PE-PLIF group was significantly less than that in the MPLIF group (*P* < 0.01). Moreover, the post-operative bedrest time was considerably shorter than that of the MPLIF group (*P* < 0.01). Additionally, the post-operative hospitalization time was significantly shorter than that in the PE-PLIF group (*P* < 0.01) (Table 2).

**Table 2:** Surgery-related outcomes in the PE-PLIF group and the MPLIF group

	PE-PLIF (*n* = 37)	MPLIF (*n* = 58)	*P*
Operation time (minutes)	204 (187, 251.5)	118 (102.5, 126.25)	< 0.001
Intraoperative blood loss (millilitre)	90 (60, 120)	200 (150, 285)	< 0.001
Post-operative bedrest time (days)	2 (1.5, 2)	5 (5, 6)	< 0.001
Post-operative hospitalization time (days)	10 (9, 10)	12 (11, 13)	< 0.001
Modified Macnab			
Excellent	36 (97.3%)	57 (98.3%)	0.630
Good	1 (2.7%)	1 (1.7%)	
Complications			
Yes	2 (5.4%)	0	0.075
No	35 (94.6%)	58 (100%)	

### Clinical efficacy index

There were no statistically significant differences in the pre-operative VAS scores for leg pain and low back pain, JOA scores or ODI scores between the PE-PLIF group and the MPLIF group (*P* > 0.05) (Table 3).

**Table 3:** Clinical outcomes of patients in PE-PLIF group and MPLIF group before and after operation

Indictor	PE-PLIF	MPLIF	*P*
VAS (leg pain)			
Pre-operative	7 (7, 8)	8 (7, 9)	0.106
Post-operative 3 days	1 (0, 2)**††	1 (1, 1)**††	0.711
Post-operative 1 week	0 (0, 0)**††	0 (0, 1)**††	0.080
Post-operative 1 month	0 (0, 0) **	0 (0, 0) **	0.645
Post-operative 6 months	0 (0, 0) **	0 (0, 0) **	0.747
Last follow-up	0 (0, 0) **	0 (0, 0) **	1.000
VAS (low back pain)			
Pre-operative	3 (1.5, 7)	3 (2.75, 5)	0.865
Post-operative 3 days	0 (0, 2) *†	3 (2, 4.25)	< 0.001
Post-operative 1 week	0 (0, 0) **	2 (1.75, 2)*†	< 0.001
Post-operative 1 month	0 (0, 0) **	0 (0, 0)**††	0.375
Post-operative 6 months	0 (0, 0) **	0 (0, 0) **	0.561
Last follow-up	0 (0, 0) **	0 (0, 0) **	0.747
JOA			
Pre-operative	8 (6.5, 9)	8 (8, 9)	0.174
Post-operative 3 days	27 (24.5, 29)**††	20 (19, 22)*†	< 0.001
Post-operative 1 week	27 (25.5, 29) **	28 (25, 29)**††	0.891
Post-operative 1 month	28 (27, 29) **	28 (27, 29) **	0.835
Post-operative 6 months	28 (28, 29) **	29 (28, 29) **	0.424
Last follow-up	29 (28, 29) **	29 (28, 29) **	0.215
ODI			
Pre-operative	16 (8, 35.5)	18.5 (14, 24.75)	0.617
Post-operative 3 days	0 (0, 9)*†	16 (11, 24.25)	< 0.001
Post-operative 1 week	0 (0, 0) **	8 (4, 10)**††	< 0.001
Post-operative 1 month	0 (0, 0) **	0 (0, 0)**††	0.369
Post-operative 6 months	0 (0, 0) **	0 (0, 0) **	0.539
Last follow-up	0 (0, 0) **	0 (0, 0) **	0.759

Compared with the group before surgery, *P < 0.05, **P < 0.01

Compared with the previous follow-up time point, †*P* < 0.05, ††*P* < 0.01

*VAS* Visual analogue scale, *JOA* Japanese Orthopaedic Association, *ODI* Oswestry Disability Index

The VAS scores for leg pain at any follow-up time point were significantly lower in the two groups than before the operation (*P* < 0.01). Compared with those 1 week after the operation and 3 days after the operation, the difference in the VAS scores for leg pain were highly significant (*P* < 0.01). There was no significant difference in the VAS scores for leg pain between 1 month after the operation and 1 week after the operation, 6 months after the operation and 1 month after the operation, and the last follow-up and 6 months after the operation (*P* > 0.05). There was no significant difference in the follow-up time points between the two groups (*P* > 0.05) (Table 3).

In the PE-PLIF group, the VAS scores for low back pain and the ODI score were lower at 3 days after the operation than those before the operation (*P* < 0.05) and significantly lower at the other follow-up time points than those before the operation (*P* < 0.01), and the JOA score was significantly higher at any follow-up time point than before the operation (*P* < 0.01). There were no significant differences in the VAS scores for low back pain, JOA scores, or ODI scores between 1 week after the operation and 3 days after the operation, 1 month after the operation and 1 week after the operation, and the last follow-up and 6 months after the operation (*P* > 0.05) (Table 3).

In the MPLIF group, 3 days after the operation, the VAS scores for low back pain and the ODI scores were not significantly different between those before the operation (*P* > 0.05), and the JOA scores were higher than those before the operation (*P* < 0.05). One week after the operation, the VAS scores for low back pain were lower than those before the operation (*P* < 0.05). The VAS scores for low back pain, JOA, and ODI scores at the other follow-up time points were highly significantly different from those before the operation (*P* < 0.01). There were significant differences in the VAS score for low back pain 1 week after the operation compared with 3 days after the operation (*P* < 0.05), and highly significant differences in the JOA and ODI scores (*P* < 0.01). There were highly significant differences in the VAS scores for low back pain and ODI scores1 month after the operation compared with 1 week after the operation (*P* < 0.01), while the JOA scores were not significantly different (*P* > 0.05). There were no significant differences in the VAS scores for low back pain or the JOA and ODI scores between 6 months after the operation and 1 month after the operation or between the last follow-up and 6 months after the operation (*P* > 0.05) (Table 3).

Compared with the two groups, the VAS scores for low back pain and the JOA and ODI scores of the PE-PLIF group were highly significantly different than those of the MPLIF group at 3 days after the operation (*P* < 0.01), and the VAS and ODI scores of the PE-PLIF group were highly significantly lower than those of the MPLIF group at 1 week after the operation (*P* < 0.01), with no significant difference at any other follow-up time points (*P* > 0.05) (Table 3).

At the last follow-up, according to the modified MacNab criteria, the excellent rate was 97.3% in the PE-PLIF group and 98.3% in the PLIF group (*P* > 0.05), with no significant difference (Table 2). In the PE-PLIF and MPLIF groups, all patients showed good results.

### Imaging results

Bridwell’s criteria were adopted to assess intervertebral fusion at 6 months after the operation. At 6 months after the operation, patients in both the PE-PLIF and MPLIF groups showed intervertebral fusion.

### Complication

In this study, major complications, such as dural tears, cerebrospinal fluid leakage, and infection, did not occur in the two groups. Two patients (5.4%) in the PE-PLIF group experienced surgical complications. Symptom secondary to contralateral nerve root compression were found in one patient. The symptoms completely resolved after ozone ablation. One patient experienced more severe pain and numbness in the right lower limb than before surgery after the PE-PLIF procedure. The symptoms were alleviated after percutaneous endoscopic discectomy. No surgical complications occurred in the MPLIF group (Table 2).

## Discussion

Leu et al. [12] reported PE-LIF technology for the first time in 1996, but its development was not rapid. Subsequently, Jacquot et al. [13] performed PE-LIF but reported a high post-operative complication rate of up to 36% and did not recommend it unless significant technical improvements were made. In recent years, with the development of spinal endoscopic devices, instruments, and techniques, this technique has regained the attention of spinal surgeons specializing in minimally invasive surgeries. Both transforaminal and posterior approaches are frequently used in PE-LIF. Percutaneous endoscopic transforaminal lumbar interbody has some shortcomings, such as limited decompression range and increased risk of damaging exiting roots because of the limited space of the Kambin triangle [14, 15]. In the posterior approach, the operational field is quite similar to that of open PLIF, and the working channel is inserted into the spinal canal during partial facetectomy and laminoplasty. The residual articular process can minimize the possibility of exiting nerve injury [16]. PE-PLIF is advantageous in that it allows decompression over a wide range and minimally stimulates the exiting nerve.

In this study, the results showed that PE-PLIF had significant advantages over MPLIF in terms of intraoperative blood loss, post-operative hospitalization time and post-operative bedrest time, but the operation time of PE-PLIF was significantly longer than that of MPLIF. In the early post-operative stage of PE-PLIF, low back pain, leg pain, and quality of life were significantly improved. Our results were similar to the results of previous studies [16, 17].

According to the study's findings, the PE-PLIF group experienced less intraoperative blood loss than the MPLIF group, which may be attributed to the following factors. First, bleeding can be prevented in advance by using radiofrequency ablation under the endoscopic field of vision, which can provide a clear surgical field of vision and reduce intraoperative bleeding [4]. Second, continuous fluid irrigation plays a vital role in controlling epidural and bone surface bleeding, which can reduce intraoperative blood loss [18]. Third, the adjacent pedicles will not be injured because a visible trephine is used for facetectomy [19], resulting in a small scope of bone resection and less bone bleeding, which is conducive to reducing intraoperative blood loss.

The operation time of PE-PLIF was significantly longer than that of MPLIF. PE-PLIF needed more time for spinal decompression and endplate preparation since smaller tools were used for facetectomy and discectomy [17, 20]. Fluoroscopy was needed to select the location for the skin incision for the channel, determine the satisfactory position of the cage in the intervertebral space, and confirm the placement of the percutaneous pedicle screws, which potentially led to a prolonged operating time due to the number of fluoroscopies. The learning curve of PE-PLIF is steep [1]. As the number of operations increases, the operation time in the later stages of PE-PLIF steadily decreases, while it is relatively long in the early stages. Fluoroscopy time and operation time can be decreased when more surgeries are performed by surgeons [21]. We have also made some process improvements to shorten the operation time of PE-PLIF, and the exact effect of these improvements needs to be observed in large control samples.

After spinal surgery, the effect of decompression is associated with the degree of recovery from leg pain [22]. Our study's findings demonstrated that post-operative leg pain was greatly reduced in both groups and almost eliminated within 1 week after the operation. This demonstrates that PE-PLIF and MPLIF both have the same decompression effect.

Our results showed that post-operative low back pain was significantly relieved in both groups. In the PE-PLIF group, the low back pain vanished 3 days after the operation, but there was no appreciable improvement in the MPLIF group at this time. In the MPLIF group, the low back pain gradually subsided 1 week after the operation and vanished completely 1 month later. The PE-PLIF group had a shorter recovery period following surgery for low back pain, which was beneficial since there was less paravertebral muscle and soft tissue damage. The PE-PLIF group had a shorter recovery period after the operation for low back pain, which was a benefit since there was less paravertebral muscle and soft tissue damage. Liu et al. [5] reported that the endoscopic spinal fusion surgery group had lower serum C-reactive protein, erythrocyte sedimentation rate, and creatine kinase levels than the conventional PLIF group. This demonstrated that there was less localized injury and less inflammation in the endoscopic spinal fusion surgery group. In the PE-PLIF group, several patients experienced severe post-operative low back pain during early surgery. Post-operative low back pain may be associated with myofascial pain syndrome (MPS). MPS is characterized by the presence of trigger points in the taut area of skeletal muscle and fascia [23, 24]. When the trigger points are activated, localized pain will appear. The symptoms can be relieved by local drug treatment [24]. Patients, who underwent early PE-PLIF surgery, experienced post-operative low back pain with tender points. The tender points were below the connecting rods. Post-operative low back pain, in the operative area, may be accompanied by the activation of myofascial trigger points. In the early cases, the fascial incision was insufficient, which led to the skeletal muscle and fascia being highly taut. The connecting rods stimulated trigger points in the taut area of the muscle and fascia, resulting in severe post-operative low back pain. The symptoms were relieved after local drug therapy. In later cases, we fully incised the fascia to reduce the degree of tautness between muscle and fascia. The occurrence of the symptom was significantly decreased.

In both groups, neurological function and the degree of limb dysfunction were dramatically improved. Three days after the operation, the degree of nerve function and limb dysfunction in the PE-PLIF group essentially reverted to normal, which may be connected to the disappearance of low back pain in the early post-operative period. The minimally invasive nature of PE-PLIF can enhance patient quality of life in the early post-operative period and encourage post-operative rehabilitation of patients, allowing patients to return to their regular social lives sooner, which is consistent with the idea of enhanced recovery after surgery (ERAS). In this study, the modified MacNab’s criteria were applied at the last follow-up, and the excellent and good rates were 97.3% in the PE-PLIF group and 98.3% in the MPLIF group. Both PE-PLIF and MPLIF showed good treatment outcomes.

It is critical to evaluate the fusion rate in patients who undergo LIF. Failed intervertebral fusion causes the cage to migrate slightly, thereby increasing the risk of endplate injury and cage subsidence/migration [25], which may affect surgical outcomes and quality of life. A study showed that at 12 months after surgery, the CT fusion rate of PE-LIF was 85.3% [26]. Insufficient intervertebral bone graft may lead to delayed intervertebral fusion [25]. In this study, all patients underwent fusion 6 months after surgery. Because the volume of autogenous bone in PE-PLIF patients was low, we implanted a mixture of autogenous bone and allograft bone with an appropriately sized cage into the intervertebral space. In PE-PLIF, the endplate was prepared under endoscopic view. The endplate cartilage could be completely removed and the risk of endplate damage by magnification of the endoscope could be reduced, which could provide a favourable fusion environment. The combination of PE-LIF and percutaneous pedicle screws placement has been widely performed and studied for the treatment of LDD [9, 21]. Percutaneous pedicle screws help achieve stability similar to that of open devices, which can maintain stability at the target level. Intervertebral fusion may benefit from appropriate interbody grafts, a favourable fusion environment, and a stable spine.

However, the PE-PLIF technique also has some limitations. The surgical indications for PE-PLIF are limited and cannot be considered when assessing the surgical candidacy of patients with severe lumbar spondylolisthesis and in need of extreme lateral decompression [16]. With the development of surgical techniques and instruments, the indications may be further extended. Fluoroscopy is needed for identifying the location for the skin incision to determine the channel, the satisfactory position of the cage in the intervertebral space, and the placement of the percutaneous pedicle screws, which lead to large radiation exposure doses for the patient and operators. In PE-PLIF, the learning curve is steep in the initial stage, the scope of the visual field under the endoscope is limited, and the operation under the endoscope is difficult [1, 21]. It is difficult for beginners to adapt to both endoscopic lumbar surgery and percutaneous pedicle screw implantation. Surgeons should perform simple endoscopic decompression in the early stage and then gradually perform PE-PLIF more frequently after gaining some experience in endoscopic technology. To increase safety and efficiency, surgeons need more advanced training to master endoscopic surgery. The development of endoscopic instruments and the accumulation of endoscopic decompression experience are conducive to mastering the learning curve of the PE-PLIF technique. PE-PLIF, combined with a navigation system and robot-assisted technology, can greatly eliminate the complexity and learning curve of endoscopic technology, is less complex and has a shorter learning curve; additionally, endoscopic technology improves the safety and accuracy of surgery, shortens the operation time, and reduces radiation exposure [27–31].

### Limitation

The limitations of this study include the following: (1) This study is a retrospective, nonrandomized controlled study, which may have partial selection bias; (2) the sample size of this study is small, and more studies with large sample sizes are needed to increase the reliability of the findings; and (3) the follow-up time of this study is short, and a longer follow-up time is needed to evaluate the long-term efficacy and complications.

## Conclusion

In our study, both PE-PLIF and MPLIF surgery were clinically effective and safe for patients with LDD. Compared with MPLIF, PE-PLIF is advantageous in that it causes less intraoperative blood loss, promotes faster recovery, and causes less tissue damage. PE-PLIF surgery can be used as an alternative treatment for single-segment LDD.

## Data Availability

The datasets used and/or analysed during the current study are available from the corresponding author on reasonable request.

## Abbreviations

LDD: Lumbar degenerative disease
LIF: Lumbar interbody fusion
LSS: Lumbar spinal stenosis
LS: Lumbar spondylolisthesis
PLIF: Posterior lumbar interbody fusion
ASD: Adjacent segment degeneration
MPLIF: Modified posterior lumbar interbody fusion
PE-LIF: Percutaneous endoscopic lumbar interbody fusion
PE-PLIF: Percutaneous endoscopic posterior lumbar interbody fusion
CT: Computed tomography
MRI: Magnetic resonance imaging
IAP: Inferior articular process
SAP: Superior articular process
rhBMP-2: Recombinant human bone morphogenetic protein-2
VAS: Visual analogue scale
JOA: Japanese Orthopaedic Association
ODI: Oswestry Disability Index
MPS: Myofascial pain syndrome
ERAS: Enhanced recovery after surgery
